# Perversely expressed long noncoding RNAs can alter host response and viral proliferation in SARS-CoV-2 infection

**DOI:** 10.2217/fvl-2020-0188

**Published:** 2020-10-27

**Authors:** Rafeed Rahman Turjya, Md. Abdullah-Al-Kamran Khan, Abul Bashar Mir Md. Khademul Islam

**Affiliations:** 1 Department of Genetic Engineering & Biotechnology, University of Dhaka, Dhaka, Bangladesh; 2 Department of Mathematics & Natural Sciences, BRAC University, Dhaka, Bangladesh

**Keywords:** COVID-19, lncRNA, long noncoding RNA, miRNA, SARS-CoV-2, viral pathogenesis

## Abstract

**Background:**

Regulatory roles of long noncoding RNAs (lncRNAs) during viral infection has become more evident in last decade, but are yet to be explored for SARS-CoV-2.

**Materials & methods:**

We analyzed RNA-seq dataset of SARS-CoV-2 infected lung epithelial cells to identify differentially expressed genes.

**Results:**

Our analyses uncover 21 differentially expressed lncRNAs broadly involved in cell survival and regulation of gene expression. These lncRNAs can directly interact with six differentially expressed protein-coding genes, and ten host genes that interact with SARS-CoV-2 proteins. Also, they can block the suppressive effect of nine microRNAs induced in viral infections.

**Conclusion:**

Our investigation determines that deregulated lncRNAs in SARS-CoV-2 infection are involved in viral proliferation, cellular survival, and immune response, ultimately determining disease outcome.

By September 2020, the confirmed cases of COVID-19 have crossed 27 million [1], making it one of the largest pandemics in modern times. Since the first reported case at Wuhan, China in December 2019 [2], this viral disease has been responsible for nearly 900,000 deaths worldwide [1], and the number is steadily rising. The causative agent behind the disease is a novel beta-coronavirus [3], which has been named SARS coronavirus 2 (abbreviated as SARS-CoV-2) due to its similarity to the earlier SARS coronavirus first detected in 2002 [4]. The rapid spread of the virus, the ever-increasing death toll and absence of a sufficient treatment strategy has affected societies and economies all over the globe.

The molecular mechanism of SARS-CoV-2 is complex and interrelated with host mechanisms, as is common with most pathogenic viruses [5]. It is possible that infected lung epithelial cells trigger innate immune pathways, leading to immune effector cells releasing high levels of chemokines and pro-inflammatory cytokines; the resultant unconfined or uncontrolled systemic inflammatory response leads to fatality [6,7]. Recent studies also indicate the virus may cause viral sepsis [8] and infection can lead to deregulation in blood coagulation [9,10]. However, no one conjecture has been able to formulate a clear and concise explanation of how the virus spreads so effectively and affects so profoundly. With emergence of more and more data, new dimensions arise as possible modes of pathogenesis and progression of infection.

Noncoding RNAs (ncRNAs), RNAs that do not code for any proteins, are key players in the regulation of gene expression and influence the interplay involved in host defense mechanisms [11]. Regulatory ncRNAs, such as microRNAs (miRNAs) and long noncoding RNAs (lncRNAs) act as important regulators of the cellular antiviral response. Consequently, viruses have been found to utilize cellular ncRNA to evade immune response and exploit cellular machinery to their advantage [12].

LncRNAs are a type of noncoding RNA having a size of more than 200 nts that can function as primary or spliced transcripts [13]. LncRNAs have become increasingly crucial in explaining cellular processes and understanding molecular progression of diseases. They may regulate gene expression through epigenetic modification of chromatin structure, transcriptional control, regulation of gene transcription via direct binding or transcription factor recruitment and post-transcriptional processing through protein-RNA interaction [14–16]. Additionally, lncRNAs may function as competing endogenous RNAs (ceRNA) [17] to function as regulators of microRNA targeting of genes involved in important pathways.

Previous studies have found lncRNAs to be involved in viral infection and subsequent host response [18–20]. A wide selection of lncRNAs get aberrantly modulated in many viral infections like- HSV, influenza, HIV etc [21]. Upon viral infections, dysregulation of cellular lncRNAs occur which in turn abnormally regulates several host processes resulting in the progression of the viral infection [20]. Apart from the general host routes, abnormalities in the expression of lncRNAs mainly affect host’s different antiviral innate immune responses, particularly interferon signaling and IFN-stimulated genes (ISGs) [20,21]. Even in SARS-CoV infection, Peng *et al.* showed that differential expression of lncRNAs could aberrantly regulate several host responses along with the innate immune signaling [22], which suggests a similar deregulation pattern of lncRNAs could also occur in SARS-CoV-2 infection.

Some attempts have been made to decipher the roles of ncRNAs in SARS-CoV-2 infection, For example, Bartoszewski *et al.* have proposed that SARS-CoV-2 could modulate host miRNA levels by acting as miRNA sponges [23]. Khan *et al.* [24] and Chow *et al.* [25] both predicted host miRNAs that target the virus. But the focus is yet to turn on to deregulated host lncRNAs and their interaction with SARS-CoV-2.

Blanco-Melo *et al.* [26] investigated the host response to SARS-CoV-2 by infecting primary human lung epithelium (NHBE) cells and A549 alveolar cell lines with the virus and performing RNA-seq analysis to identify differentially expressed (DE) genes. But no such study yet concluded the possible outcomes of the deregulated lncRNAs in SARS-CoV-2. In our present study we have identified lncRNAs that are differentially expressed in SARS-CoV-2 infected cell’s transcriptome compared with uninfected cells, and then correlated the putative effects of the deregulated lncRNAs in the tug-of-war between SARS-CoV-2 and the host. We have also investigated the possible aftermaths of lncRNA deregulation in COVID-19 disease pathobiology.

## Materials & methods

### Identification of differentially expressed lncRNAs

Raw FastQ reads of RNA-Seq performed by Illumina were extracted from GEO database (GEO accession GSE147507) [27]. The experimental data included independently assessed biological triplicates of primary human lung epithelium (NHBE) cells, which were either mock treated or infected with SARS-CoV-2 and cultured for 24 h. The reads were mapped with TopHat (TopHat v2.1.1 with Bowtie v2.4.1) [28]. Using the latest version of human reference genome GRCh38, as downloaded from UCSC database [29], short reads were uniquely aligned, allowing at best two mismatches. Sequences that were exact matches to multiple regions with equal quality were discarded to avoid bias [30]. The reads not mapped to the genome were utilized by mapping against the transcriptome. The latest version of Ensembl gene model [31] (version 99, as extracted from UCSC) was utilized for this process. After completion of mapping, we used SubRead package featureCount v2.21 [32] to calculate the absolute read abundance, leading to read count (r.c.) for each of the Ensembl genes. For the subsequent differential expression (DE) analysis, we used DESeq2 v1.26.0 with R v3.6.2 (2019-07-05) [33] that employs a model based on negative binomial distribution. To avoid false positives, transcripts with at least ten reads annotated in at least one of the samples used in this study were considered.

### PPI network construction

From RNA-seq data analysis, 638 DE protein-coding genes were identified. These were combined with the recently reported 332 SARS-CoV-2 protein interactors [34] retrieved from BioGRID [35] to build a protein–protein interaction (PPI) network. Edge information for this network were extracted from the STRING [36] database, where only interactions with high confidence (score >0.700) were selected and retrieved. A network file was prepared in simple interaction format to be visualized using Cytoscape v3.7.2 [37].

### Retrieval of RNA–RNA interactions & lncRNA functions

We retrieved previously established RNA–RNA interactions between the DE lncRNAs and other RNAs from NPInter v4.0 [38]. Among the interacting RNAs, we searched for DE protein-coding genes and viral protein-interactor genes. Functions of the DE lncRNAs were retrieved from LncBook [39]. The viral proteins that bind to lncRNA–interacting host proteins were also identified.

### Identification of virally-induced miRNAs

miRwayDB [40] provided information about human miRNAs that were previously identified as involved in viral-mediated diseases. Additionally, information about host miRNAs involved in viral infection as identified by Girardi *et al.* [41] and miRNAs involved in viral acute respiratory infections as described by Leon-Icaza *et al.* [42] were retrieved. These miRNAs were manually curated for their involvement in viral pathogenesis and possible connection to SARS-CoV-2.

### Extraction of microRNA targets

Gene targets for the curated set of miRNAs were retrieved from experimentally validated miRNA–gene interactions from miRTarBase [43] and filtered for the DE protein-coding genes. DIANA-LncBase v3 [44] was used to retrieve miRNAs that target the DE lncRNAs, considering only high-confidence interactions. Combining the lists led to identification of the miRNAs that target both DE protein-coding genes and lncRNAs. The set of upregulated target genes for each miRNA were analyzed using NetworkAnalyst 3.0 [45] tools for functional over-representation and network enrichment. miRNAs that mostly targeted upregulated genes were finally selected.

### Construction of biological networks

Networks depicting the interactions between DE lncRNAs, DE genes, viral proteins and curated miRNAs were built using Cytoscape v3.7.2 [37]. Gradient coloring scheme was used to depict the change in expression of DE genes.

## Results

### lncRNAs differentially modulated in SARS-CoV-2 infection

**Figure 1.**
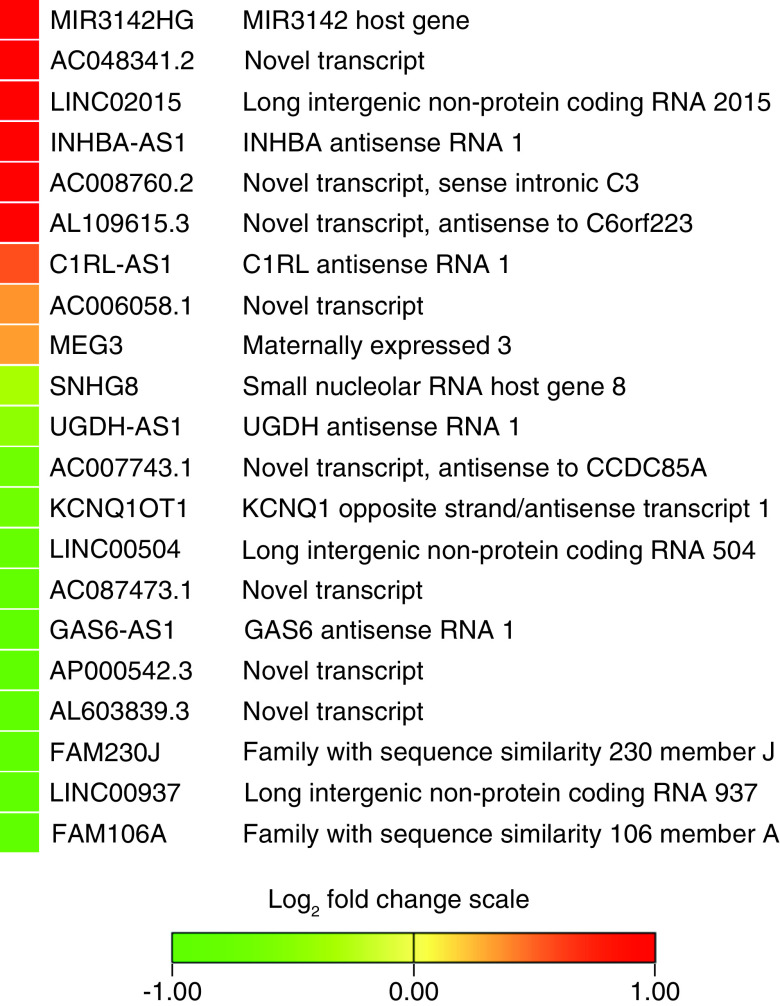
Differentially expressed long noncoding RNAs in SARS-CoV-2 infected primary human lung epithelium (NHBE) cells. Analyzing the RNA-seq data, nine lncRNAs were found to be upregulated, while twelve were downregulated. Gene expressions are presented in log2 fold change (compared with uninfected control cells) value color coded heatmap. Color toward red indicates more upregulation and color toward green indicates further downregulation, while yellow color indicates absence of differential expression.

To investigate the probable deregulation of lncRNAs in SARS-CoV-2 infection, first we have analyzed the 24 h postinfection transcriptome data of SARS-CoV-2 infected NHBE cells. Analyzing the RNA-seq data led to identification of 687 DE genes (Supplementary file 1). Intriguingly among those DE genes, we discovered 21 lncRNA genes, nine of them upregulated while 12 were downregulated (Figure 1).

### lncRNAs interact with several differentially modulated proteins in SARS-CoV-2 infected cells

**Figure 2.**
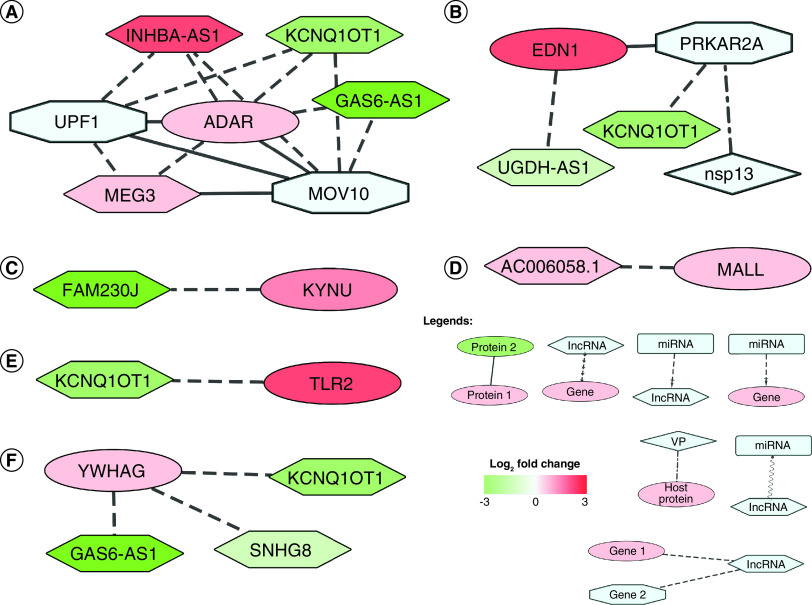
Differentially expressed long noncoding RNAs interact with differentially expressed protein-coding genes. **(A)** Upregulated *ADAR* interacts with downregulated *KCNQ1OT1* and *GAS6-AS1*, and upregulated *MEG3* and *INHBA-AS1*; it also interacts with UPF1 and MOV10. **(B)** Upregulated *EDN1* interacts with downregulated *UGDH-AS1* and Nsp13 interactor PRKAR2A. **(C)** *KYNU* mRNA interacts with downregulated *FAM230J*. **(D)** Upregulated *MALL* interacts with upregulated *AC006058.1*. **(E)** Upregulated *TLR2* interacts with downregulated *KCNQ1OT1*. **(F)** YWHAG protein interacts with downregulated *KCNQ1OT1*, *SNHG8* and *GAS6-AS1*. Node shape legends: ellipse: DE gene; hexagon: lncRNA; diamond: VP; octagon: viral protein interactor; rectangle: microRNA; types of edges are- dot-dash: VP-host protein interaction; dash: lncRNA–gene interaction; dash-arrow: miRNA–gene interaction; solid: PPI; sine wave: ceRNA; separate arrows: alternate target. Log2 fold change color scale is as depicted in Figure 1. ceRNA: Competing endogenous RNA; DE: Differentially expressed; lncRNA: Long noncoding RNA; PPI: Protein–protein interaction; VP: Viral protein.

We now sought to elucidate the putative effects of these deregulated lncRNAs in SARS-CoV-2 infection. In order to achieve that, we built a network of the deregulated lncRNAs along with their interacting protein coding target genes. Among the differentially expressed protein-coding genes, six were found to interact with the DE lncRNAs (Table 1). Among them, there are direct RNA–RNA interactions for four genes and RNA–protein interactions for the rest (Figure 2). These lncRNA interacting protein coding genes have potential roles during the viral infections (Table 1).

**Table 1.** Differentially expressed genes, their encoded protein’s functions and associated interacting long non-coding RNAs.

Gene name	Protein function	lncRNA interactor	Interaction Type	Ref.
*ADAR*	*ADAR* works as a regulator in RIG-I/MDA5 mediated induction of IFN-α/β pathways and controls innate immune response against dsRNA in the cell. It exerts an antiviral effect on HCV, but works in favor of VSV, MV, HDV and type 1 HIV-1.	*KCNQ1OT1 GAS6-AS1* *MEG3* *INHBA-AS1*	Protein–RNA	[46–54]
*EDN1*	Endothelin-1 is an endothelium-derived vasoconstrictor peptide that belongs to the endothelin/sarafotoxin family. The peptide works as a potent vasoconstrictor and its cognate receptors are therapeutic targets in the treatment of pulmonary arterial hypertension. It is involved in downstream GPCR-controlled signaling.	*UGDH-AS1*	RNA–RNA	[55,56]
*KYNU*	*KYNU* gene encodes Kynureninase enzyme, which catalyzes the cleavage of L-kynurenine and L-3-hydroxykynurenine into anthranilic acid and 3-hydroxyanthranilic acid, respectively. Among its functional pathways are NAD metabolism and tryptophan utilization.	*FAM230J*	RNA–RNA	[57,58]
*MALL*	*MALL* gene encodes an element of the machinery for raft-mediated trafficking in endothelial cells. The encoded protein, a member of the MAL proteolipid family, predominantly localizes in glycolipid- and cholesterol-enriched membrane (GEM) rafts. It interacts with caveolin-1 and is involved in cholesterol homeostasis.	*AC006058.1*	RNA–RNA	[59,60]
*TLR2*	*TLR2* encodes the toll-like receptor 2 protein, a fundamental protein in the pathways involved with pathogen recognition and activation of innate immunity. A cell-surface protein that can form heterodimers with other TLR family members, it can recognize conserved molecules derived from microorganisms known as PAMPs. Activation of TLRs by PAMPs switches on signaling pathways that modulate the host’s inflammatory response. *TLR2* acts via *MYD88* and *TRAF6*, leading to NF-κB activation, cytokine secretion and inflammatory response. This gene may also promote apoptosis in response to bacterial lipoproteins.	*KCNQ1OT1*	RNA–RNA	[61–65]
*YWHAG*	*YWHAG* encodes the 14-3-3 protein gamma, an adapter protein implicated in the regulation of a large spectrum of both general and specialized signaling pathways. It is induced by growth factors in human vascular smooth muscle cells, and is also highly expressed in skeletal and heart muscles, suggesting an important role for this protein in muscle tissue. Among its interactors are RAF1 and PKC, which link it to various signal transduction pathways. The protein is involved in apoptosis and PI3K-Akt signaling pathway. *YWHAG* was identified as a direct target of miR-181b-3p, a metastasis activator which downregulated *YWHAG* to promote Snail-induced EMT in breast cancer cells. MiR-182 was found to suppress esophageal squamous cell carcinoma cell proliferation and metastasis via regulating *YWHAG*	*KCNQ1OT1* *SNHG8* *GAS6-AS1*	Protein–RNA	[66–73]

EMT: Epithelial-mesenchimal transformation; HDV: Hepatitis delta virus; MV: Measles virus; GPCR: G protein-coupled receptors; PAMP: Pathogen-associated molecular pattern; TLR: Toll like receptor; VSV: Vesicular stomatitis virus.

Upregulated *ADAR* interacts directly with downregulated *KCNQ1OT1* and *GAS6-AS1*, and upregulated *MEG3* and *INHBA-AS1* (Figure 2A). ADAR protein also interacts with UPF1 and MOV10 – two proteins that are also interactors of other DE lncRNAs. *EDN1* is found to be upregulated in SARS-CoV-2 infection, which interacts directly with downregulated *UGDH-AS1* (Figure 2B). EDN1 protein can also interact with PRKAR2A, a protein that interacts with *KCNQ1OT1* and viral protein Nsp13. *KYNU* mRNA interacts directly with *FAM230J*, which was downregulated (Figure 2C) [74]. *MALL*, a gene found upregulated in SARS-CoV-2 infection, interacts directly with *AC006058.1*, which was also upregulated (Figure 2D). Upregulated *TLR2* interacts directly with downregulated *KCNQ1OT1* (Figure 2E). YWHAG protein interacts directly with *KCNQ1OT1*, *SNHG8*, *GAS6-AS1*, all downregulated (Figure 2F).

### Protein interactors of SARS-CoV-2 viral proteins also interacted with DE lncRNAs

**Figure 3.**
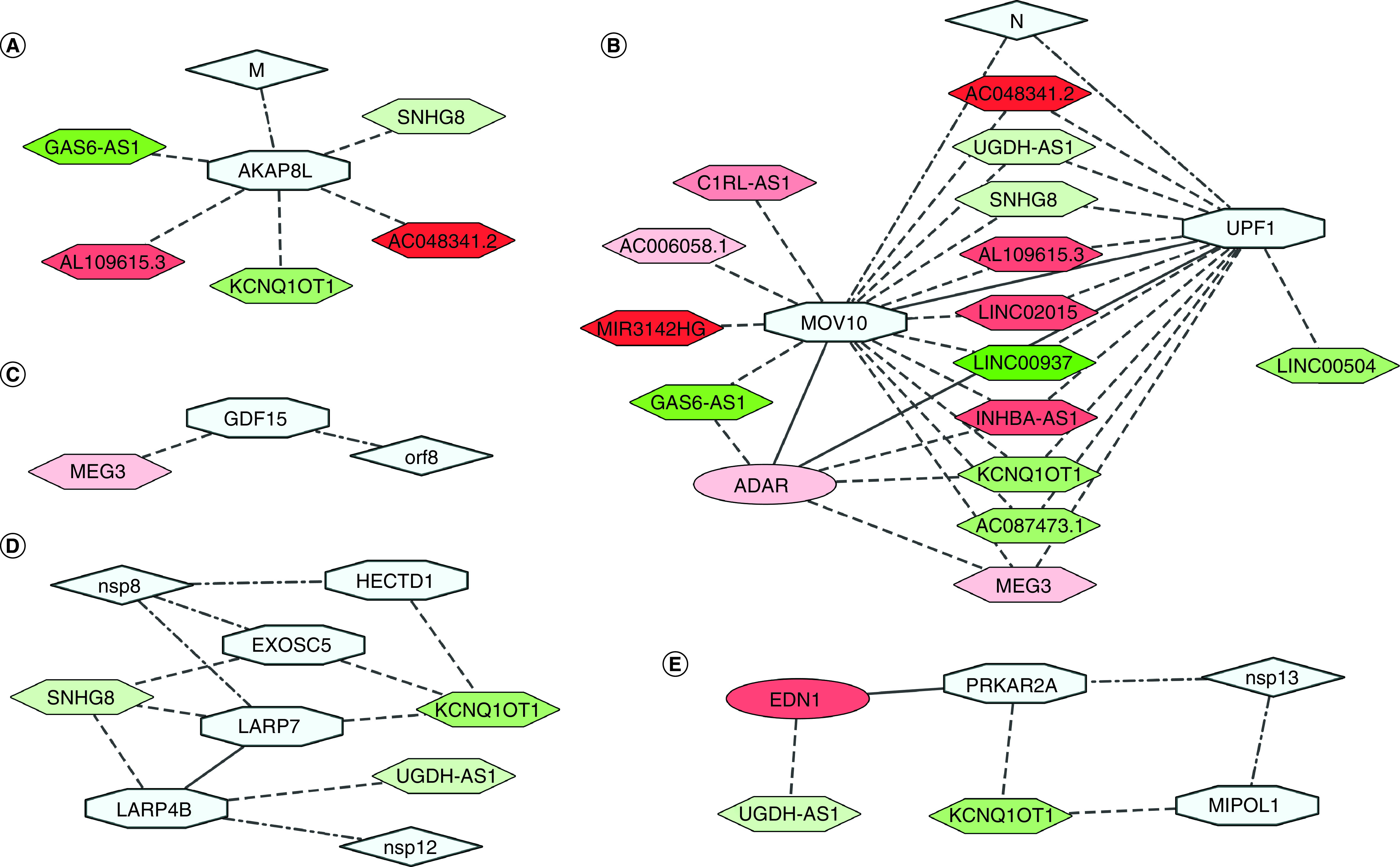
Differentially expressed long noncoding RNAs interact with SARS-CoV-2 protein interacting genes. **(A)** M protein interactor AKAP8L interacts with downregulated *KCNQ1OT1*, *GAS6-AS1*, *SNHG8*, *AC048341.2* and upregulated *AL109615.3*. **(B)** Among N protein interactors, UPF1 and MOV10 proteins interacts directly with multiple DE lncRNAs, and each other. Both also interact with ADAR protein. **(C)** *MEG3* can stimulate expression of *GDF15*, an interactor of viral protein orf8. **(D)** Nsp8 interactors EXOSC5 and LARP7 proteins interact with downregulated *KCNQ1OT1* and *SNHG8*; *HECTD1* mRNA interacts with *KCNQ1OT1*. Nsp12 interactor LARP4B protein interacts with downregulated *UGDH-AS1* and *SNHG8*, and also with LARP7. **(E)** Nsp13 interactors *MIPOL1* and *PRKAR2A* interact with downregulated *KCNQ1OT1*; PRKAR2A also interacts with *UGDH-AS1* interactor EDN1. Color codes, node and edge notations are similar as Figure 2. DE: Differentially expressed; lncRNAs: Long noncoding RNAs.

Gordon *et al.* investigated possible protein–protein interactions (PPI) between SARS-CoV-2 proteins and host proteins and identified 332 high-confidence interactions [34]. We retrieved these interactions from BioGRID [35], which are provided in supplementary file 2. We wanted to link whether these interacting proteins can also interact with the DE lncRNAs in SARS-CoV-2 infection. Subsequently, we constructed a network with the DE lncRNAs and their interacting host proteins, which also bind with viral proteins. Among these 332 proteins, we found ten such proteins that also interact with the DE lncRNAs (Table 2). Among them, seven proteins interacted at the protein–RNA level, whereas the rest were RNA–RNA interactions. SARS-CoV-2 proteins M (Membrane), N (Nucleocapsid), Nsp8, Nsp12, Nsp13, and ORF8 were found to interact with these host proteins (Figure 3).

**Table 2.** SARS-CoV-2 interactor host proteins and associated interacting long non-coding RNAs.

Gene name	Protein function	SARS interactor protein	lncRNA interactor	Interaction type	Ref.
*AKAP8L*	AKAP8L (A-kinase anchor protein 8-like) protein probably plays a role in CTE-mediated gene expression by association with DHX9 by increasing nuclear unspliced mRNA export. In EBV infected cells, it may target PRKACA to nuclear sites containing EBNA-LP (an EBV protein) to modulate transcription from specific promoters. In synergy with DHX9, it can activate the CTE-mediated gene expression of type D retroviruses. In case of HIV-1 infection, it is involved in the DHX9-promoted annealing of host tRNA (Lys3) to viral genomic RNA as a primer in reverse transcription.	M	*KCNQ1OT1* *GAS6-AS1* *SNHG8* *AC048341.2* *AL109615.3*	Protein–RNA	[75–78]
*EXOSC5*	EXOSC5 is a noncatalytic component of the RNA exosome complex, which has 3′ to 5′ exoribonuclease activity and participates in a multitude of cellular RNA processing and degradation events.	Nsp8	*KCNQ1OT1* *SNHG8*	Protein–RNA	[79]
*GDF15*	*GDF15* is a member of the GDNF family which binds to GDNF family receptor α-like (GFRAL) protein, a transmembrane receptor exclusively expressed in the hind brain. The protein is expressed in a broad range of cell types, acts as a pleiotropic cytokine and is involved in the stress response program of cells after cellular injury. Increased protein levels are associated with disease states such as tissue hypoxia, inflammation, acute injury and oxidative stress.	ORF8	*MEG3*	RNA–RNA	[80–83]
*HECTD1*	HECTD1 is an E3 ubiquitin-protein ligase which accepts ubiquitin from an E2 ubiquitin-conjugating enzyme in the form of a thioester and then directly transfers the ubiquitin to targeted substrates and mediates ‘Lys-63’-linked polyubiquitination of HSP90AA1, which leads to its intracellular localization and reduced secretion. The protein is involved in class I MHC mediated antigen processing and presentation and innate immune system.	Nsp8	*KCNQ1OT1*	RNA–RNA	[84]
*LARP4B*	*LARP4B* encodes a La-module containing factor that can bind AU-rich RNA sequence directly and promotes mRNA accumulation and translation. It was deemed a candidate tumor suppressor gene in glioma, as it was consistently decreased in human glioma stem cells and cell lines compared with normal neural stem cells. *LARP4B* overexpression strongly inhibited cell proliferation by inducing mitotic arrest and apoptosis and *CDKN1A* and *BAX* were upregulated.	Nsp12	*UGDH-AS1* *SNHG8*	Protein–RNA	[85,86]
*LARP7*	LARP7 works as a negative transcriptional regulator of polymerase II genes, acting by means of the 7SK RNP system. This snRNP complex inhibits a cyclin-dependent kinase, positive transcription elongation factor b, which is required for paused RNA polymerase II at a promoter to begin transcription elongation.	Nsp8	*KCNQ1OT1* *SNHG8*	Protein–RNA	[87,88]
*MIPOL1*	*MIPOL1* encodes a coiled-coil domain-containing protein, which may function as a tumor suppressor.	Nsp13	*KCNQ1OT1*	RNA–RNA	[89]
*UPF1*	UPF1 protein, an RNA-dependent helicase and ATPase, is required for NMD of mRNAs containing premature stop codons. It is recruited to mRNAs upon translation termination by release factors to stalled ribosomes together with the SMG1C protein kinase complex to form the transient SURF (SMG1-UPF1-eRF1-eRF3) complex.	N	*AC048341.2* *LINC00937* *GAS6-AS1* *KCNQ1OT1* *UGDH-AS1* *SNHG8* *AC087473.1* *AL109615.3* *INHBA-AS1* *MEG3* *LINC02015* *MIR3142HG* *AC006058.1* *C1RL-AS1*	Protein–RNA	[90–92]
*MOV10*	MOV10 is a 5′ to 3′ RNA helicase contributing to UPF1 mRNA target degradation by translocation along 3′ UTRs and is involved in miRNA-mediated gene silencing by the RISC. It plays a role for both miRNA-mediated translational repression and miRNA-mediated cleavage of complementary mRNAs by RISC. UPF1-MOV10 is involved in antiviral activity through both NMD pathway and IFN induction.	N	*LINC00504* *AC048341.2* *LINC00937* *GAS6-AS1* *KCNQ1OT1* *UGDH-AS1* *SNHG8* *AC087473.1* *AL109615.3* *INHBA-AS1* *MEG3* *LINC02015*	Protein–RNA	[93–98]
*PRKAR2A*	PRKAR2A is the cAMP-dependent protein kinase type II-alpha regulatory subunit, which works as the regulatory subunit of the cAMP-dependent protein kinases involved in cAMP signaling in cells.	Nsp13	*KCNQ1OT1*	RNA–RNA	[89]

CTE: Constitutive transport element; GDNF: Glial cell-derived neurotropic factor; NMD: Nonsense-mediated decay; RISC: RNA-induced silencing complex.

AKAP8L protein, an M protein interactor, interacts directly with downregulated *KCNQ1OT1*, *GAS6-AS1*, *SNHG8*, *AC048341.2* and upregulated *AL109615.3* (Figure 3A). Among N protein interactors, UPF1 and MOV10 proteins interact directly with DE lncRNAs, and also interact with each other (Figure 3B). UPF1 protein interacts with downregulated *AC048341.2*, *LINC00937*, *GAS6-AS1*, *KCNQ1OT1*, *UGDH-AS1*, *SNHG8* and *AC087473.1*, and upregulated *AL109615.3*, *INHBA-AS1*, *MEG3*, *LINC02015*, *MIR3142HG*, *AC006058.1* and *C1RL-AS1*. MOV10 protein interacts with *LINC00504*, *AC048341.2*, *LINC00937*, *GAS6-AS1*, *KCNQ1OT1*, *UGDH-AS1*, *SNHG8* and *AC087473.1*, which were downregulated, and *AL109615.3*, *INHBA-AS1*, *MEG3* and *LINC02015*, which were upregulated. Both UPF1 and MOV10 proteins also interact with ADAR protein, an interactor of *KCNQ1OT1*, *GAS6-AS1*, *MEG3* and *INHBA-AS1* (Figure 3B). *MEG3* can stimulate expression of *GDF15* by enhancing p53 binding to the *GDF15* gene promoter [80], while GDF15 protein can be targeted by viral orf8 (Figure 3C).

Among Nsp8 interactors, EXOSC5 and LARP7 proteins interact with *KCNQ1OT1* and *SNHG8*, both downregulated; another interactor *HECTD1* mRNA interacts directly with *KCNQ1OT1* (Figure 3D). Among Nsp12 protein interactors, LARP4B protein interacts with *UGDH-AS1* and *SNHG8*, both downregulated, and with LARP7 (Figure 3D). Among Nsp13 interactors, *MIPOL1* and *PRKAR2A* both have RNA–RNA interactions with downregulated *KCNQ1OT1*. PRKAR2A also interacts with EDN1, which interacts with *UGDH-AS1* (Figure 3E).

### Upregulated targets of virally-induced microRNAs might be protected by competing lncRNAs

**Figure 4.**
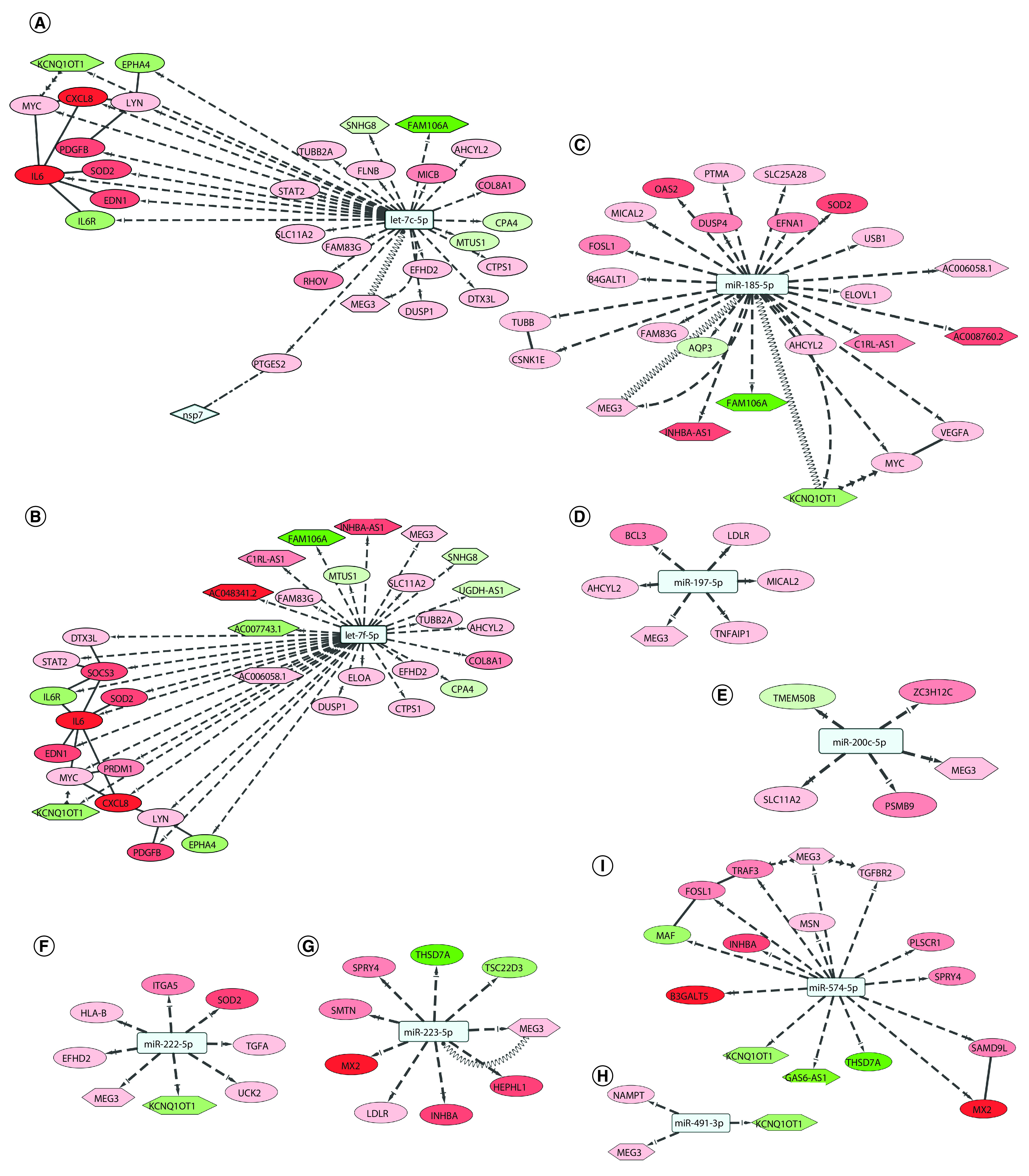
Potential virally induced miRNAs and their targets. **(A)** miRNA let-7c can target 21 upregulated genes and four differentially expressed long non-coding RNAs; *MEG3* can act as a competing endogenous RNA. **(B)** miRNA let-7f can target 20 upregulated genes and nine DE lncRNAs; *MEG3* can act as a ceRNA. **(C)** miR-185-5p can target 17 upregulated genes and seven DE lncRNAs; *MEG3* and *KCNQ1OT1* can act as ceRNAs. **(D)** miR-197-5p can target five upregulated genes and lncRNA *MEG3*. **(E)** miR-200c-5p can target three upregulated genes and lncRNA *MEG3*. **(F)** miR-222-5p can target six upregulated genes and lncRNAs *KCNQ1OT1* and *MEG3*. **(G)** miR-223-5p can target six upregulated genes and lncRNA *MEG3*, which can act as a ceRNA. **(H)** miR-491-3p can target upregulated *NAMPT* and lncRNA *MEG3*. **(I)** miR-574-5p can target ten upregulated genes and lncRNAs *MEG3*, *KCNQ1OT1* and *GAS6-AS1*. Color codes, node and edge notations are similar as Figure 2. ceRNA: Competing endogenous RNA; DE: Differentially expressed; lncRNAs: Long noncoding RNAs.

ceRNA hypothesis illustrates that lncRNAs and other RNA molecules harboring miRNA response elements can suppress the expression and biological function of each other by competing for miRNAs that can bind to the complementary regions, thus regulating miRNA-mediated gene silencing of the target genes [17,99]. To reveal if such networks exists in SARS-CoV-2 infected cells, a list of miRNAs induced in viral infection was curated from miRwayDB database [40], Girardi *et al.* [41], and Leon-Icaza *et al.* [42]. Among them, few were found to target genes upregulated in the cells, which were apparently not targeted as DE lncRNAs were targeted simultaneously (Figure 4). The names and functions of all identified target genes of the selected miRNAs that were differentially expressed in the infected cells are provided in supplementary file 3.

In SARS-CoV-2 infection, miRNA let-7c might target 21 upregulated genes and can also target *MEG3*, *KCNQ1OT1*, *SNHG8* and *FAM106A* lncRNAs (Figure 4A). The 21 upregulated targets of let-7c-5p are significantly enriched for KEGG pathways like – Kaposi’s sarcoma-associated herpesvirus infection, and JAK-STAT signaling pathway (data not shown). Among the target genes, PTGES2 protein was found to interact with SARS-CoV-2 Nsp7 protein (Figure 4).

miRNA let-7f can target 20 of the upregulated genes in SARS-CoV-2 infection. It can also target lncRNAs *AC006058.1*, *AC007743.1*, *AC048341.2*, *C1RL-AS1*, *FAM106A*, *INHBA-AS1*, *KCNQ1OT1*, *MEG3* and *SNHG8* (Figure 4B). The 20 upregulated targets of the let-7f-5p are involved in pathways like Kaposi’s sarcoma-associated herpesvirus infection-related, JAK-STAT signaling, and hepatitis B related signaling (data not shown).

miR-185-5p targets 17 upregulated genes, but also targets lncRNAs *AC006058.1*, *AC008760.2*, *C1RL-AS1*, *FAM106A*, *INHBA-AS1*, *KCNQ1OT1* and *MEG3* (Figure 4C). Among the 17 upregulated targets of the miR-185-5p miRNA, most significantly enriched KEGG pathways were MAPK signaling pathway, and Wnt signaling pathway (data not shown).

miR-197-5p, miR-200c-5p, miR-222-5p and miR-223-5p target five, three, six and six upregulated genes, respectively (Figure 4D–G). They can all target lncRNA *MEG3* while miR-222-5p can additionally target *KCNQ1OT1.* The six upregulated targets of the miR-222-5p are involved in phagosome, and PI3K-Akt signaling pathway (data not shown).

miR-491-3p targets upregulated *NAMPT* gene, but also targets *MEG3* lncRNA (Figure 4H). miR-574-5p targets ten upregulated genes, but also targets lncRNAs *MEG3*, *KCNQ1OT1* and *GAS6-AS1* (Figure 4I). The ten upregulated targets of the miR-574-5p are found significantly enriched in biological process/pathways like TGF-β signaling pathway, IL-17 signaling pathway and apoptotic process (data not shown).

### Involvement of lncRNA may modulate important cellular pathways

Cell survival can be beneficial for continued viral replication and growth, but apoptosis is also necessary for further spread. Pathways related to cell survival are targeted by virally-induced microRNAs. IL-6/JAK/STAT3 signaling acts in favor of cell survival [100]. Upregulation of IL-6 during certain viral infections may promote virus survival and/or exacerbation of clinical disease [101]. Among let-7c-5p targets and let-7f-5p targets, four upregulated genes, *IL6*, *MYC*, *PDGFB* and *STAT2* belong to JAK-STAT signaling pathway. *KCNQ1OT1* can act as a sponge for *MYC* gene, a target of miR-185-5p along with these miRNAs (Figure 4C [102]). Also, both let-7c and let-7f target IL-6, and absence of this interaction may lead to cell transformation progressing from initial inflammation, implying cell survival [103].

Wnt signaling pathway is known to regulate apoptosis through a variety of mechanisms [104] and it can respond to viral infections through modulation of β-catenin stabilization [105]. *MYC*, *CSNK1E* and *FOSL1* belong to Wnt signaling pathway and are targeted by miR-185-5p. *FOSL1* is also targeted by miR-574-5p (Figure 4I).

Activation of PI3K-Akt signaling is a known viral strategy to delay apoptosis and prolong viral replication, as seen in acute and persistent infection [106]. Three upregulated genes, *EFNA1*, *MYC* and *VEGFA*, targeted by miR-185-5p, and two upregulated genes targeted by miR-222-5p, *ITGA5* and *TGFA*, belong to PI3K-Akt signaling pathway (Figure 4). Also, miR-185 was found to inhibit cell proliferation and induce apoptosis by targeting *VEGFA* directly in von Hippel-Lindau-inactivated clear cell renal cell carcinoma [107].

NF-κB signaling pathway is involved in upregulating various pro-inflammatory genes that encode cytokines and chemokines [108]. It is a major regulator of antiviral response, and NF-κB activation pathways are manipulated by viruses to avoid cellular mechanisms that eliminate the infection [109,110]. Two upregulated gene targets of let-7c-5p and let-7f-5p, *CXCL8* and *LYN*, and two upregulated targets of miR-574-5p, *TRAF3* and *TGFBR2* is involved in NF-κB signaling pathway (Figure 4). *MEG3* can act as a sponge to promote upregulation of *TRAF3* [111] and *TGFBR2* [112]. *BCL3*, a target of miR-197-5p, is involved in regulation of cell proliferation and participates in NF-κB signaling pathway (Figure 4D).

MAPK pathway positively regulates virus replication in diverse group of viruses [113]. Three upregulated gene targets of let-7c-5p, *MYC*, *PDGFB* and *DUSP1*, belong to MAPK signaling pathway (Figure 4A). *LDLR*, involved with cholesterol homeostasis, is targeted by miR-197-5p and miR-223-5p (Figure 4).

## Discussion

Long noncoding RNAs are regulators and modulators of complex cellular pathways and viral infection is no exception [19,20]. Dysregulation of lncRNA expression and interaction, either directly with viral RNA, or indirectly with host RNA, affect the progression of viral infection [12]. LncRNAs can work as a sponge for miRNA, bind protein as a competitive inhibitor, inhibit PPI, influence post-translational modification, or affect the activity of target protein. They can modulate viral life cycle, regulate innate immune response or assist adaptive immunity [18,21]. In our exploration of the deregulated lncRNA in SARS-CoV-2 infected NHBE cells, all of these functionalities come to fore. In our study, we find lncRNA-interacting DE protein-coding genes and SARS-CoV-2 protein interactors to be involved in various pathways. Both RNA–RNA and RNA–protein interactions were present, indicating a complex interplay between the components involved. Additionally, these lncRNAs may perform as sponges to protect upregulated genes that would otherwise be targeted by virally-induced miRNAs. Our findings shed light on the possible role of DE lncRNAs in specific processes involved with viral infection, proliferation and cellular response.

SARS-CoV-2 Nsp7-Nsp8-Nsp12 proteins interact to form a multi-subunit RNA-synthesis complex, where Nsp12 works as the RNA-dependent RNA polymerase. EXOSC5, interactor of Nsp8 protein, is a noncatalytic component of the RNA exosome complex. RNA exosome complex is involved in 3′ processing of various stable RNA species and is crucial for RNA quality control in the nucleus [114], thus Nsp8 binding may be fundamental to diminishing the capacity of exosome to act against viral mRNAs. Antiviral drug remdesivir has been predicted to target and disassemble this complex [115,116]. Interaction of EXOSC5 protein with *KCNQ1OT1* and *SNHG8* can modulate this interaction. The putative RNA–RNA interaction between upregulated *AC006058.1* and *MALL* mRNA, a gene involved in cholesterol homeostasis and membrane trafficking, can exert an influence in maturation of SARS-CoV-2, an enveloped virus. *FAM230J*, which has no reported function, is downregulated in the infected cells. Its absence may activate the upregulated *KYNU*, which is involved in metabolite biosynthesis pathways, through lack of RNA–RNA interaction.

Host RNA-binding proteins that interact with SARS-CoV-2 proteins are involved in regulating viral transcription and mRNA stability. This is evident through the interaction of SARS-CoV-2 nucleocapsid (N) protein with UPF1 and MOV10 proteins. In case for murine hepatitis virus, a model coronavirus, the N protein carries out this interaction to inhibit nonsense-mediated decay (NMD) of viral mRNAs containing multiple stop codons, thus favoring viral mRNA transcription. NMD pathways recognized cytoplasmic viral mRNAs as a substrate for degradation, but viral replication induced the inhibition of the NMD pathway through N protein [117]. In human T-lymphotropic virus type 1-infected cells, viral protein tax bound to components of NMD pathways, including UPF1, to inhibit the process [118]. SARS-CoV-2 Membrane (M) protein interacts with AKAP8L, which assists in viral infection progression through favoring transcription. Both LARP7 (nsp8 interactor) and LARP4B (nsp12 interactor) are RNA-binding proteins involved in RNA transcription regulation. Interaction of these RNA-binding proteins with the DE lncRNAs may be crucial for viral mRNA transcription and stability against cellular defense.

ADAR can be regarded as the ‘Editor-in-Chief’ of innate immunity against viral infection [119]. *MEG3* was found to act as a biomarker and regulate cell functions by targeting *ADAR* in colorectal cancer. The cells overexpressing *MEG3* exhibited increased *ADAR* expression, and downregulation of *MEG3* was found in colorectal cancer tissues, cell lines and serum [120]. In the infected cells, *MEG3* upregulation may lead to *ADAR* overexpression, which could have been favorable to the virus, as evidenced in influenza A [121]. *INHBA-AS1* upregulation in virus-infected cells may also contribute to cell survival through interaction with *ADAR*, as evidenced in gastric cancer [122] and oral squamous cell carcinoma [123]. As the role of *ADAR* as a controlling element in cellular response to viral infection is paramount, its regulation by *MEG3* and interactions with lncRNAs in SARS-CoV-2 infected cells may influence progression of the disease.

Cell survival can be both beneficial to virus and be inhibitory. Viruses need the cell to survive for a certain period to undergo replication, but they also need apoptosis to exit the cell and infect others. Thus, cell survival and apoptosis becomes a key indicator of pathogenesis. Among lncRNAs, downregulated *SNHG8* has possible link to apoptosis and cell death [124], as does *KCNQ1OT1* [125–128]. Unlike both, downregulation of *GAS6-AS1* may be linked with cell proliferation and survival [129,130]. All three interact with YWHAG, which is involved in signal transduction. IL-6/JAK/STAT3 signaling, Wnt signaling pathway, and PI3K-Akt signaling, involved in cell survival, are also protected by probable ceRNA function by lncRNA.

Cellular response to viral infection can make or break the progression of viral life cycle. Innate immune response is inevitable, but the complex interactions underlying its activation and effect may have been the target of lncRNAs. HECTD1 is an E3 ubiquitin-protein ligase that interacts with Nsp8. As it is involved in class I MHC mediated antigen processing and presentation and innate immune system, the binding may lead to modulation of that response. In case of *HECTD1* mRNA, absence of RNA–RNA interaction with downregulated *KCNQ1OT1* lncRNA can facilitate the response. *PTGES2* gene was upregulated and also found to interact with SARS-CoV-2 Nsp7 protein. As it is involved in innate immune system and signaling pathways, this binding may exert an indirect influence. TLR2, as part of the innate immune response, has been connected to antiviral action against multiple viral infections [131–134]. *KCNQ1OT1*, the lncRNA having putative RNA–RNA interaction with *TLR2*, was found to attenuate sepsis-induced myocardial injury via regulating miR-192-5p/*XIAP* axis. Downregulation of the lncRNA advanced cardiac injury by allowing miR-192-5p to target *XIAP* [135]. *XIAP* functions in the inhibition of apoptosis, whereas *TLR2* is known to promote apoptosis. The downregulation of *KCNQ1OT1* in infected cells thus will promote apoptosis, in accordance with its interaction with overexpressed *TLR2* mRNA. Virally-induced microRNAs can also be involved in targeting pathways related with inflammation and host defense. Response by NF-κB signaling pathway against the viral infection is probably protected by lncRNA against inhibition by these miRNAs.

DE lncRNAs also had probable involvement in regulating cellular processes, including signaling. The downregulation of *UGDH-AS1* can be related to *EDN1* overexpression, which can be crucial in vasoconstriction, leading to severe symptoms, as seen in SARS-CoV-2 infections [136]. SARS-CoV-2 Nsp13 protein works as a viral helicase and interacts with MIPOL1 and PRKAR2A. Function of MIPOL1 is unclear, whereas PRKAR2A is involved in cAMP-mediated signaling, which is the probable target for modulation by Nsp13. The RNA–RNA interactions of downregulated *KCNQ1OT1* with the mRNAs of these two genes may have implications on viral infection progression. Additionally, MAPK pathway is probably regulated through lncRNA involvement.

Growing evidence indicates that the ceRNA regulation mechanism plays a role in disease progression and drug efficiency [137–140]. We checked if any of the DE lncRNAs in the infected cells were exerting such effects to ensure a particular miRNA does not downregulate a gene. In turn, the upregulated genes play a crucial role in viral replication, disease progression and immune response. Although some of these miRNAs also target a few downregulated genes, their decrease in expression may be controlled by separate mechanisms. There were previous instances where some of these miRNAs were blocked by a specific lncRNA that was deregulated in the infected cells. For instance, *KCNQ1OT1* was found to regulate *Rab14* expression, a target of miR-185-5p, in oral squamous cell carcinoma cells as a ceRNA [141]. *MEG3* aggravated palmitate-induced insulin resistance by regulating miR-185-5p/*Egr2* axis as a ceRNA in insulin-resistant hepatocytes [142]. Additionally, miRNA let-7c-5p was found to negatively regulate *NLRC5* expression in ethanol-induced hepatic injury, where *MEG3* functioned as a ceRNA against the miRNA [143]. *MEG3* acted as an endogenous sponge to suppress the function of miR-223 and to increase *NLRP3* expression and enhance endothelial cell pyroptosis in ox-LDL-treated human aortic endothelial cells [144]. *KCNQ1OT1* plays the role of a ceRNA to regulate *MYC*, an upregulated target of let-7c-5p, let-7f-5p and miR-185-5p [102]. We can deduce that these lncRNAs can be involved in a similar role in infected cells.

## Conclusion

Our findings explore the molecular footprint of SARS-CoV-2 infection from the lens of lncRNAs. The integrated analysis has linked multiple actors in the complex interplay of molecules in exacerbating the infection and identified possible drug targets. We trace the involvement of lncRNAs with cellular behavior in this situation and illuminate their role in cell survival, viral replication and immune defense. The lethality and swift transmission of this virus is entwined with the deregulated cellular environment, and lncRNA regulation is crucial for understanding its parameters. Our results could provide insights for scheming some novel RNA therapeutics during the lacking of effective cure option.

## Future perspective

SARS-CoV-2 has wreaked havoc across the world within a short period. This virus may persist in the population and assume a seasonal nature. The dysregulation in cellular level is ultimately responsible for the disease, and lncRNAs are crucial pieces of the puzzle. The lncRNAs can control cellular response and determine disease outcome. Our elucidation of these molecular connections can deepen our understanding of the virus and the nature of its infection. Effective treatment methods of the future will certainly rely on this knowledge.

**Summary points**

- 21 long noncoding RNAs (lncRNAs) are dysregulated in SARS-CoV-2 infected lung epithelial cells.
- These lncRNAs are involved in cell survival and regulation of gene expression.
- Six differentially expressed genes interact with these lncRNAs.
- Ten proteins that interact with SARS-CoV-2 viral proteins also interact with these lncRNAs.
- These protein-coding genes function in cellular signaling, metabolism, immune response and RNA homeostasis.
- The lncRNAs can act as competing endogenous RNAs for virally induced microRNAs.
- Effect of these microRNAs center on host defense and cell survival.
- The interactions affect cellular processes underlying disease progression.
